# Functional MR imaging beyond structure and inflammation—radiographic axial spondyloarthritis is associated with proteoglycan depletion of the lumbar spine

**DOI:** 10.1186/s13075-020-02312-x

**Published:** 2020-09-17

**Authors:** Daniel B. Abrar, Christoph Schleich, Styliani Tsiami, Anja Müller-Lutz, Karl Ludger Radke, Neela Holthausen, Miriam Frenken, Matthias Boschheidgen, Gerald Antoch, Johanna Mucke, Philipp Sewerin, Juergen Braun, Sven Nebelung, Xenofon Baraliakos

**Affiliations:** 1 Department of Diagnostic and Interventional Radiology, University Düsseldorf, Medical Faculty, 40225 Düsseldorf, Germany; 2 Rheumazentrum Ruhrgebiet Herne, Ruhr University Bochum, Claudiusstr. 45, 44649 Herne, Germany; 3 Policlinic and Hiller Research Unit of Rheumatology, UKD, Heinrich Heine University Düsseldorf, Moorenstrasse 5, 40225 Düsseldorf, Germany

**Keywords:** Ankylosing spondylitis, Magnetic resonance imaging, gagCEST, Spine, Rheumatic diseases, Spondyloarthropathy

## Abstract

**Background:**

To compare the glycosaminoglycan (GAG) content of lumbar intervertebral disks (IVDs) of patients with ankylosing spondylitis (AS) and healthy volunteers and to investigate the association of GAG depletion and disease-related clinical and imaging features.

**Methods:**

Lumbar spines of 50 AS patients (mean age 50 ± 10.5 years) and 30 age-matched volunteers were studied with 3-T magnetic resonance imaging (MRI) and conventional radiographs (CR). The MRI protocol included high-resolution morphological sequences and the compositional GAG chemical exchange saturation transfer imaging technique (gagCEST). Morphological images were analyzed by three raters for inflammatory activity, fat deposition, disk degeneration, and structural changes on CR. Clinical and serological measures included the Bath AS Disease Activity (BASDAI) and Bath AS Function (BASFI) Indices and C-reactive protein (CRP) levels. GagCEST values of both groups were compared using a linear mixed model. Kendall-Tau correlation analyses were performed.

**Results:**

GagCEST values were significantly lower in AS patients (2.0 ± 1.7%) vs. healthy volunteers (2.4 ± 1.8%), *p* = 0.001. Small, yet significant correlations were found between gagCEST values and CRP levels (*τ* = − 0.14, *p* = 0.007), BASFI (*τ* = − 0.18, *p* < 0.001) and presence of syndesmophytes (*τ* = − 0.17, *p* = 0.001). No significant correlations were found with BASDAI, inflammation, and fat deposition MRI scores.

**Conclusions:**

Lumbar spines of r-AS patients undergo significant GAG depletion, independently associated with syndesmophyte formation, functional disability, and increased serological inflammation markers. Beyond establishing a pathophysiological role of the cartilage in AS, these findings suggest that gagCEST imaging may have an adjunct confirmatory role in the assessment of disease-related pathological MRI findings in axial spondyloarthritis.

**Trial registration:**

3980 (https://studienregister.med.uni-duesseldorf.de)

## Introduction

Ankylosing spondylitis (AS), also called radiographic spondyloarthritis (r-axSpA), as the prototype disease of all axSpA, is a chronic inflammatory disease that predominantly affects the sacroiliac joints (SIJ) and the spine [1]. It leads to back pain, restricted mobility, and, if left untreated, to ankylosis and severe functional disability [2, 3]. As of today, magnetic resonance imaging (MRI) is a cornerstone imaging technique for identification and may also be helpful in treatment monitoring of axSpA [4]. This is reflected by the inclusion of MRI of the SIJ into the Assessment of SpondyloArthritis Society (ASAS) classification criteria [5]. However, MRI of the spine was not included in these criteria due to the lack of specificity when compared to degenerative spinal lesions [6]. Nonetheless, in current clinical and research practice, MRI of both the SIJ and the spine is widely used due to its non-invasiveness, superior soft tissue contrast, high spatial resolution, functional capability, and absence of ionizing radiation.

On the other hand, new bone formation is a key feature of chronic stages of axSpA and contributes to the burden of the disease and functional disability beyond mere inflammation-related symptoms [7–9]. Earlier studies demonstrated that joint remodeling in patients with r-axSpA is characterized by incipient cartilage loss, subchondral infiltration of fibrous tissue, and later on eventual formation of new bone [10, 11]. In an earlier study, our group demonstrated the feasibility of glycosaminoglycan chemical exchange saturation transfer imaging (gagCEST) of the lumbar intervertebral disks (IVD) in a pilot study of 9 patients with axSpA [12]. GagCEST imaging is a compositional MRI technique that relies on the chemical exchange of water protons between GAG and bulk water molecules. For the induction of a CEST effect, a pool of solute protons is saturated by a frequency-specific radiofrequency (RF) pulse at different offset frequencies around the water resonance and then transferred to the bulk water pool via chemical exchange, which consequently reduces its signal. The signal is then used to quantify the CEST effect at the GAG-specific frequency range (0.9–1.9 ppm) by analysis of the magnetization transfer ratio asymmetry (MTR_asym_), which correlates with the underlying GAG concentration of the given IVD [13–15].

The aim of this study was to systematically assess the association of GAG depletion in lumbar IVDs with structural and inflammatory imaging features as well as clinical and serological reference measures in a larger patient population of r-axSpA patients and controls.

Building on preliminary evidence that suggested a role for advanced imaging techniques in the evaluation of r-axSpA [12, 16, 17], we hypothesized that (a) lumbar IVDs of patients with r-axSpA contained less GAG than a group of age-matched volunteers and (b) that GAG depletion was associated with disease-related acute and chronic inflammation and structural changes of the adjacent vertebrae as well as functional limitations.

## Methods

### Study population

Fifty patients with active r-axSpA according to the ASAS classification criteria [5] (mean age 50 ± 10.5, range 25–69 years) and mean disease duration of 13 ± 11 years (range, 0–40 years) were prospectively recruited. R-axSpA was defined as definitive radiographic sacroiliitis on the sacroiliac joint conventional radiography according to the New York criteria [18, 19]. At the time of recruitment, 44/50 patients received biological disease-modifying antirheumatic drugs (bDMARDs), while the other 6 patients were not treated with any permanent medication. In addition, 30 volunteers (mean age 47 ± 13.5, range 30–76 years) were included as a control group. The exclusion criteria for all participants were prior spine surgery, a body mass index < 18.5 or > 30 kg/m^2^, radiculopathy, known disk extrusion, congenital spine deformities, and being underage. For the control group, the exclusion criteria were expanded to chronic lower back pain (LBP), acquired spine deformities, and chronic inflammatory diseases affecting the musculoskeletal system.

Written informed consent was acquired from all participants before the initiation of the study. The study was approved by the local ethical committee (Ethical Committee, Medical Faculty, University of Düsseldorf, Germany, study number 5087R).

The characteristics of the study population are given in Table 1.

**Table 1:** Demographic, clinical, serological, and functional information of the study population

	Patients	Volunteers
Age [years]	50 ± 10.5	47 ± 13.5
Sex [female/male]	14/36	15/15
Disease duration [years]	9 ± 7	na
CRP level [mg/dL]	0.75 ± 0.76	na
BASDAI [0–10]	5.13 ± 1.84	na
BASFI [0–10]	5.36 ± 2.15	na
Medication	bDMARD [*n* = 44] None [*n* = 6]	na

Data are given as means ± standard deviations. Only for the radiographic axial spondylarthritis (r-axSpA) patients were data on disease durations, CRP levels, and functional scores, i.e., Bath Ankylosing Spondylitis Disease Activity and Bath Ankylosing Spondylitis Function Indices (BASDAI and BASFI), available. *na* not available; *CRP* C-reactive protein, normal range 0–0.5 mg/dL; *bDMARD* biological disease-modifying drug

### Imaging studies

MRI studies of the lumbar spine of all participants were performed on a clinical 3-T scanner (Magnetom Prisma, Siemens Healthineers, Erlangen, Germany) with a 32-channel body and a 24-channel spine matrix coil (both Siemens Healthineers) in the supine position.

All MRI examinations included morphological and compositional sequences. Morphological imaging comprised T1-weighted (T1w), T2-weighted (T2w), and short tau inversion recovery (STIR) sequences in the sagittal orientation, while compositional imaging included gagCEST and water saturation shift referencing (WASSR) sequences in the sagittal orientation. The detailed sequence parameters are given in Table 2.

**Table 2:** Detailed magnetic resonance imaging (MRI) sequence parameters

Sequence					
	STIR	T2w TSE	T1w TSE	gagCEST	WASSR
Imaging plane	Sagittal	Sagittal	Sagittal	Sagittal	Sagittal
TE/TR [ms]	57/3800	95/3500	9.5/650	5.1/10	5.1/10
Flip angle [°]	150	160	150	10	10
Slice thickness [mm]	4	4	4	5	5
FoV [mm × mm]	300 × 300	300 × 300	300 × 300	300 × 300	300 × 300
Pixel size [mm × mm]	0.8 × 0.8	0.7 × 0.7	0.7 × 0.7	1.6 × 1.6	1.6 × 1.6
Number of slices	15	15	15	1	1

Imaging plane, echo and repetition time (TE/TR), flip angle, slice thickness, field of view (FoV), pixel size, and number of slices are given for all sequences (short tau inversion recovery, T2-weighted turbo spin-echo (T2w TSE), T1w TSE, gagCEST, and WASSR)

Additionally, standard conventional radiographs (CR) of the lumbar spines (Th12–S3) in anterior-posterior (ap) and lateral projections were obtained after the recruitment.

### Image analysis

All images were blinded to the participants’ diagnoses and demographics and were then independently analyzed by two radiologists (DBA and CS) with long-standing experience in musculoskeletal imaging. In case of discrepant findings, consensus was established with the assistant opinion of a third experienced rater (SN).

The lumbar IVD segments L1/L2–L5/S1 of all participants were scored individually on sagittal T2w images according to the Pfirrmann classification [20]. Briefly, the Pfirrmann classification is based on five grades and allows dichotomization of non-degenerative (grades 1 and 2) and degenerative IVDs (grades 3–5) on the basis of intensity and structure of the nucleus pulposus (NP), its distinction from the annulus fibrosus (AF), and disk height.

Further, each lumbar segment was separately analyzed according to the Spondyloarthropathies Research Consortium of Canada (SPARCC) score, which is an MRI index for scoring inflammation of the spine [21]. To this end, each lumbar discovertebral unit (DVU) was divided into four quadrants: upper anterior, lower anterior, upper posterior, and lower posterior endplate. The presence of “acute inflammation/bone marrow edema” was assessed based on the high signal intensity on STIR images. For each DVU, three consecutive slices were selected demonstrating the most pathological slices for that particular segment. On each slice, the imaging feature “bone marrow edema” was scored present or absent resulting in a maximum score of 12 per DVU. In addition, we evaluated all lumbar segments for the presence of “fatty depositions” (as a sign of chronic inflammation) by proceeding in the same manner. The presence of “fatty deposition” was assessed based on the high signal intensity on T1w and T2w images.

Additionally, as a radiographic score, all anterior vertebral corners (VC) of the lumbar segments were evaluated (score 0–2) on lateral CR projections for the presence of syndesmophytes (score 1) or bridging syndesmophytes (score 2).

GagCEST analyses were in line with the standard post-processing practice as published previously [22, 23]. Before gagCEST analysis, motion correction was performed for both CEST and WASSR datasets using a diffeomorphic registration approach incorporated into a dedicated software (fMRLung, Siemens Healthineers) as published before [24].

As in earlier studies [25, 26], WASSR data were used to correct *B*_0_ field inhomogeneities by the WASSR maximum-symmetry algorithm with the calculation of a pixel-wise frequency offset curve. These offset-corrected CEST curves divided by the signal without pre-saturation (*S*_0_) were defined as the so-called *z*-spectrum (*Z* (*ω*)). The maximum frequency offset of each *z*-spectrum was Δ*ω* = 3 ppm. Next, we used the magnetization transfer asymmetry (MTR_asym_) (defined as MTR_asym_ (Δ*ω*) = Z(−Δ*ω*) − *Z* (Δ*ω*)) for the evaluation of the gagCEST effect [25]. MTR_asym_ maps were calculated using the average value of MTR_asym_ in the GAG-specific range of Δ*ω* = 0.9–1.9 ppm. MTR_asym_ values are given in percentage. For the analysis of the CEST effect, regions of interest (ROIs) were defined based on a customized in-house script implemented in Matlab (MATLAB, R2018a, The MathWorks, Inc., MA, USA) that automatically detects lumbar IVD segments.

Then, all ROIs were automatically placed in the lumbar IVDs by an in-house written script for the Matlab software. The disk segmentation was based on Bayes classification to divide bone and ligaments from disk cartilage [27]. All automatically placed ROIs were visually confirmed for the correct position by a board-certified radiologist (CS). No ROIs had to be manually corrected. In total, nine individuals with 45 IVDs had to be excluded from the gagCEST analysis due to excessive motion artifacts (6 r-axSpA, 3 volunteers). For the sake of readability, MTR_asym_ values are referred to as gagCEST values.

### Clinical evaluation

For every patient, the Bath Ankylosing Spondylitis Disease Activity Index (BASDAI) and Bath Ankylosing Spondylitis Function Index (BASFI) [28, 29] were assessed. In addition, C-reactive protein levels [mg/dL] were determined in all patients.

### Statistical analysis

The SPSS software (IBM, version 22, Armonk, NY, USA) was used for all statistical analyses performed by KLR and DBA. For descriptive analysis, mean gagCEST values of the AF and the NP ± standard deviation, median, and range (minimum-maximum) were calculated for volunteers and patients. For the comparison of gagCEST values between both cohorts and between AF and NP, a multivariable statistical analysis was performed using a linear mixed model (LMM). The model included a subject-specific random intercept, the factors healthy volunteer/patient, age, gender, and the interaction of these factors. The LMM was fitted using a restricted maximum likelihood approach (REML) [30]. Based on this final model, the mean differences of gagCEST values were calculated and evaluated for significance. For correlation analyses, the Kendall Tau correlation coefficient *τ* was calculated. Correlation strengths were graded as suggested by Cohen [31]: small (< 0.3), moderate (0.3–0.5), and large (> 0.5). *p* values < 0.05 were considered significant.

## Results

### Clinical scores: BASDAI, BASFI, and serum CRP levels

Patient characteristics and serological, clinical, and functional data are summarized in Table 1. Overall, patients showed active disease, with a mean BASDAI of 5.1 ± 1.8 and mean CRP levels of 0.8 ± 0.8 mg/dL, and also high levels of functional impairment, with a mean BASFI of 5.4 ± 2.2.

### Morphological analysis

**Fig. 1:**
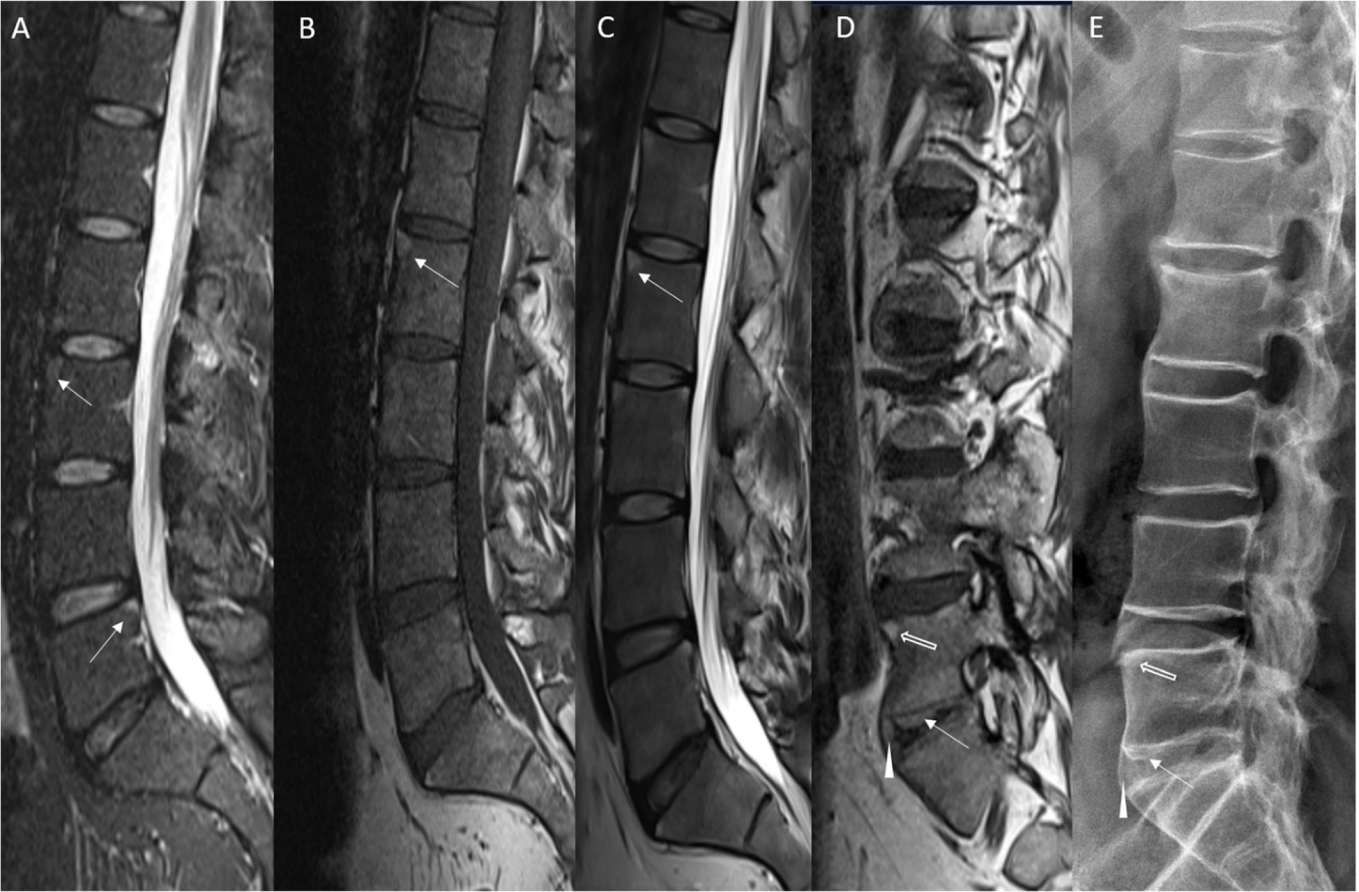
Multi-modality representation of typical imaging findings in ankylosing spondylitis (AS). Morphological MRI findings (**a**–**d**) and radiographical findings (**e**) are demonstrated. **a**–**c** Sagittal short tau inversion recovery (STIR, **a**), T1-weighted (T1w, **b**), and T2-weighted (T2w, **c**) images of the lumbar spine (T12–S2) of a 31-year-old male with r-axSpA. Typical disease-related changes are acute inflammation of vertebral corners (**a**) that are visible as multi-segmental focal signal hyperintensities of the anterior and posterior corners of vertebral endplates (white arrows in **a**). Signs of chronic inflammation, i.e., fatty infiltration, of the vertebral endplate corners are detected as signal hyperintensities in T1w and T2w images (white arrows in **b** and **c**). **d**, **e** Sagittal T1w image (obtained laterally at the height of the neuroforaminal openings) and lateral radiographic projection of the lumbar spine of a 46-year-old male with AS. Here, chronic inflammation at a vertebral endplate corner is visible as a focal signal hyperintensity (block arrow in **d**) or focal sclerosis (block arrow in **e**) of the upper anterior corner of the vertebral body of L5. New bone formations can be seen as bridging syndesmophytes (arrowheads) and as transdiskal ankylosis (white arrows)

Morphological imaging findings for the presence of syndesmophytes and fatty depositions, and according to the Pfirrmann classification, and SPARCC are shown in Table 3. Typical disease-related MR imaging features are shown in Fig. 1.

**Table 3:** Analysis of morphological and compositional imaging features of lumbar intervertebral disks (IVDs)

Score		Segment						*p* value
		L1/2	L2/3	L3/4	L4/5	L5/S1	Overall	
gagCEST values [%]	Patients	2.7 ± 1.7	2.1 ± 1.7	1.9 ± 1.6	1.8 ± 1.4	1.9 ± 2.0	2.0 ± 1.7	**0.001**
	Volunteers	3.1 ± 1.7	2.2 ± 1.6	2.1 ± 1.6	2.4 ± 1.7	2.1 ± 2.3	2.4 ± 1.8	
Pfirrmann Score [1–5]	Patients	2.4 ± 0.7	2.5 ± 0.8	2.4 ± 0.7	2.5 ± 0.6	2.7 ± 0.8	2.5 ± 0.7	0.119
	Volunteers	2.1 ± 0.5	2.3 ± 0.7	2.4 ± 0.6	2.4 ± 0.6	2.6 ± 0.7	2.4 ± 0.6	
SPARCC acute inflammation [0–12]	Patients	1 ± 2.4	0.4 ± 1.3	0.2 ± 1	0.6 ± 1.6	0.6 ± 2.3	0.6 ± 1.8	0.318
Fatty depositions [0–12]	Patients	4.3 ± 3.9	3.4 ± 2.8	3.9 ± 3.2	4.2 ± 3.1	3.8 ± 3.1	3.9 ± 3.2	0.723
Syndesmophytes [0–2]	Patients	1.3 ± 1.3	1.2 ± 1.2	1.3 ± 1.1	1.2 ± 1.2	1.4 ± 1.4	1.3 ± 1.2	0.894

The mean imaging measures as a function of the study cohort (i.e., patient and volunteer) and intervertebral disk segment level. Data are means ± standard deviations. The mean values of the glycosaminoglycan chemical exchange saturation transfer (gagCEST) values were compared with a linear mixed model comprising a subject-specific random intercept. Overall, the mean values of the Pfirrmann grading, the SPARCC, presence of fatty depositions, and syndesmophytes of the patient and the control group were compared by the Kruskal-Wallis test. *p* values < 0.05 were considered significant. MRI scores were Pfirrmann scores, Spondyloarthropathy Research Consortium of Canada (SPARCC), and the presence of fatty deposition (chronic inflammation of vertebral corners). The presence of syndesmophytes was scored by CR

The distribution of IVDs according to the Pfirrmann classification were as follows: total study population—grade 1 [*n* = 0], grade 2 [*n* = 285], grade 3 [*n* = 99], grade 4 [*n* = 14], and grade 5 [*n* = 2]; r-axSpA patients—grade 1 [*n* = 0], grade 2 [*n* = 180], grade 3 [*n* = 58], grade 4 [*n* = 10], and grade 5 [*n* = 2]; volunteers—grade 1 [*n* = 0], grade 2 [*n* = 105], grade 3 [*n* = 41], grade 4 [*n* = 4], and grade 5 [*n* = 0].

All scores were equally distributed between the lumbar segments, i.e., no significant differences were found between the evaluated lumbar segments at any given score (Table 3).

### Comparative analysis of gagCEST values: NP vs. AF and NP vs. Pfirrmann grading

In all IVDs, the NP showed significantly higher gagCEST values than the AF (AF 1.1 ± 1.3%, NP 2.2 ± 1.8%, *p* < 0.001). GagCEST values were significantly affected by the morphological degeneration of the IVD as assessed by the Pfirrmann score. NPs of IVDs with Pfirrmann grades ≤ 2, i.e., non-degenerated IVDs, had significantly higher gagCEST values than NPs of IVDs with Pfirrmann grades ≥ 3, i.e., degenerated IVDs—NPs (Pfirrmann grades ≤ 2) 2.7 ± 1.7% and NPs (Pfirrmann grades ≥ 3) 0.7 ± 0.9%; *p* = 0.003. No significant differences were found between gagCEST values of the NPs of IVDs of the different lumbar segments in patients and volunteers (L1/2 2.1 ± 1.7%, L2/3 2.2 ± 1.7%, L3/4 2.0 ± 1.7%, L4/5 2.1 ± 1.5%, L5/S1 1.9 ± 2.1%, *p* = 0.06).

### Comparative analyses of gagCEST values: patients vs. volunteers

Mean gagCEST values of r-axSpA patients and volunteers as well as the comparative analysis are presented in Table 3.

**Fig. 2:**
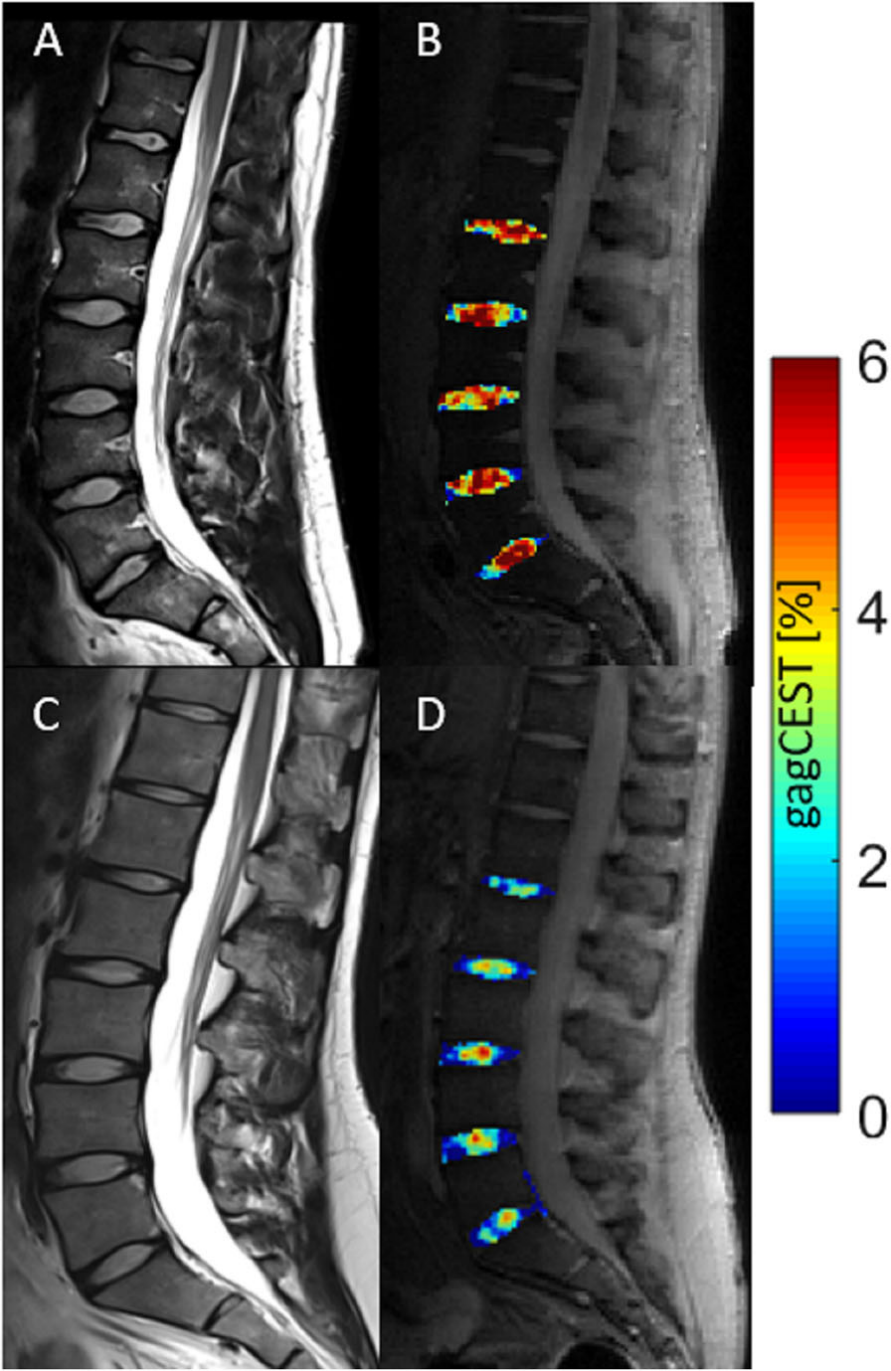
Representative morphological and compositional imaging findings of the lumbar spine of a volunteer (**a**, **b**) and a patient with r-axSpA (AS; **c**, **d**). **a**, **c** Sagittal T2-weighted images show the absence of gross morphological signs of degeneration, in particular of the intervertebral disks (IVDs). **b**, **d** Sagittal gagCEST images with overlaid colored gagCEST maps for visualization of the GAG content. High GAG content as indicated by high gagCEST values is illustrated in red, while low GAG content is indicated in blue. Notably, this patient with r-axSpA demonstrated lower GAG contents than this volunteer despite non-degenerated IVDs in both individuals

Over all segments, gagCEST values of the NP of lumbar IVDs of r-axSpA patients were significantly lower than of volunteers (patients 2.0 ± 1.7%, control 2.4 ± 1.8%, *p* = 0.001). GagCEST maps of representative patients and volunteers confirmed these findings (Fig. 2).

No significant differences were found between the gagCEST values of treated and non-treated patients (treated 2.1 ± 1.8; non-treated 2.1 ± 1.24; *p* = 0.904).

### Correlation analyses

The correlations of compositional cartilage measures, i.e., gagCEST values, with serological (CRP level), clinical/functional (BASDAI and BASFI), and imaging (SPARCC, fatty depositions, syndesmophytes) scores of lumbar IVDs are presented in Table 4.

**Table 4:** Correlation analyses of gagCEST values of lumbar IVDs and serological (CRP level), clinical/functional (BASDAI and BASFI), and imaging scores. *p* values < 0.05 were considered significant and are given in bold. Please refer to Table 3 for an explanation of the abbreviations

		Kendall Tau correlation coefficient *τ*	*p* value
gagCEST values vs.	CRP levels	− 0.14	**0.007**
	BASDAI	0.04	0.400
	BASFI	− 0.18	**< 0.001**
	SPARCC acute inflammation	0.06	0.285
	Fatty depositions	− 0.09	0.055
	Syndesmophytes	− 0.17	**0.001**

Significant, yet small and negative correlations were found between gagCEST values and CRP levels (*τ* = − 0.14 *p* = 0.007), BASFI (*τ* = − 0.18, *p* < 0.001), and syndesmophytes (*τ* = − 0.17, *p* = 0.001). No significant correlations were found between gagCEST values and BASDAI, SPARCC, and fatty depositions.

## Discussion

The most important findings of this study were that lumbar IVDs of patients with r-axSpA had significantly lower gagCEST values, i.e., a lower GAG content, than those of an age-matched control group and that these compositional changes were associated with variable structural changes on the level of the patient and the spine. More specifically, the lumbar IVDs’ GAG content was associated with serological CRP levels, functional BASFI scores, and presence of syndesmophytes, while no association was observed with acute or chronic inflammation scores or patient-reported BASDAI scores.

Anecdotical evidence suggested that the composition of IVDs changed in patients with axSpA as compared to healthy volunteers [12]. Building on the preliminary findings, our study provides substantial evidence that compositional changes of the lumbar spine are associated with numerous structural and inflammatory changes in a larger and homogenous cohort of patients with r-axSpA, which is considered the prototypical axSpA disease. GAG depletion is an established sign of early cartilage changes that precedes morphologically visible damage [32, 33]. Interestingly, since the 1950s, research has indicated that cartilage loss plays a significant role in the pathophysiology of r-axSpA, too [34, 35]. Also, more recent studies have highlighted the affection of the joint cartilage in the disease course. In histological studies, Bleil et al. demonstrated that cartilage degradation as shown by cartilage thinning, increased chondrocyte apoptosis, and proteoglycan loss promoted by the invasion of the subchondral bone are hallmark changes of joint remodeling in r-axSpA [10, 36, 37]. Furthermore, they demonstrated that cartilage involvement in r-axSpA differed from OA, especially regarding the affection of the subchondral plate [10]. According to other studies, the cartilage layer of the joints is the primary site for inflammation and necessary for its induction [11]. Our results confirm these histological findings in the area of the vertebral bodies, as decreased GAG contents of morphologically unremarkable lumbar IVDs were observed in patients with r-axSpA. It was hypothesized that the development of syndesmophytes is preceded by cartilage loss [8]. These syndesmophytes are not only a mere sign of long-standing inflammation, but are the main cause of irreversible functional disability and independent from inflammation-related stiffness and pain [7]. As the prevention of radiographic disease progression is a major treatment goal in r-axSpA [38], a better understanding of syndesmophyte formation and earlier detection of bone proliferations seems beneficial and a promising diagnostic target. It might be beneficial to evaluate cartilaginous structures for subtle changes that eventually lead to syndesmophyte formation, potentially allowing for earlier diagnosis and treatment. Therefore, considering our findings, gagCEST imaging could be of great interest for expanded disease stratification and treatment monitoring.

Our study demonstrates significant, yet small negative correlations between the GAG contents of lumbar IVDs and CRP levels, the functional BASFI score, and the presence of syndesmophytes. In contrast, no correlations were found between GAG contents and the BASDAI scores as an established patient-reported outcome of disease activity, or the SPARCC score with its focus on acute inflammation, or the presence of fatty depositions as a sign of chronic inflammatory changes of the vertebral endplates. Hence, overall, weak correlations were found for GAG content and structural changes of the spine and functional disability, but not for inflammatory imaging features and inflammation-related pain and stiffness. Considering the abovementioned concepts, it seems plausible that the GAG contents demonstrated significant correlations with structural changes and functional disability as opposed to no significant correlations with inflammatory changes and inflammation-related symptoms. Against this background, the earlier study by Roos and Dahlberg has to be considered, which demonstrated physical activity might lead to anabolic GAG remodeling [39]. In their study, patients at risk of osteoarthritis of the knee joint displayed an increase in GAG in their articular cartilage after completing a 4-month exercise routine. Considering that patients with r-axSpA are prone to physical inactivity due to the disease, the opposite effects of catabolic GAG depletion are likely.

When interpreting our results, some limitations need to be mentioned. First, gagCEST imaging of the cartilage still requires histological validation. Because of obvious ethical considerations, we could not perform IVD biopsies for histological analyses. Second, the disease duration of our patient cohort was quite heterogeneous ranging from 0 to 40 years, which might be the reason for increased statistical variability, yet it is fully reflective of the clinical reality. Also, most of our r-axSpA patients were treated by bDMARDs with largely well-controlled symptomatology. Nonetheless, future studies should include a more homogenous study population, in particular in terms of disease duration to establish the potential value of compositional assessment of the spine across variable disease durations. Third, we only evaluated lumbar, but not thoracic IVDs, which are usually considered in established axSpA imaging scores, such as the mSASSS. However, as of today, gagCEST imaging is especially prone to movement artifacts, such as breathing, and may not be implemented at the thoracic level in the foreseeable future. Further, the proportion of females was higher in the control than in the patient group, which potentially affected the results, since anecdotical evidence suggests a higher proteoglycan content of lumbar IVDs in females than in males [26]. However, since the factor “gender” was included in the LMM that was used for our statistical analysis, we consider the differences in gender distribution only a minor limitation.

## Conclusions

In conclusion, we found a significantly lower GAG content in lumbar IVDs of patients with r-axSpA compared to the healthy control group. Lower GAG contents were associated with structural alterations of the spine such as syndesmophyte formation, and functional disabilities but not with inflammatory changes of the vertebral endplates or inflammation-related pain and stiffness. Beyond establishing a pathophysiological role of the cartilage in r-axSpA, these findings suggest that—once substantiated by further studies—gagCEST imaging may have an adjunct diagnostic role in the assessment of disease severity and treatment effects in spondyloarthritis.

## Data Availability

The datasets used and/or analyzed during the current study are available from the corresponding author on reasonable request.

## Abbreviations

AS: Ankylosing spondylitis
ASAS: Assessment of SpondyloArthritis International Society
axSpA: Axial spondyloarthritis
BASDAI: Bath Ankylosing Spondylitis Disease Activity Index
BASFI: Bath Ankylosing Spondylitis Function Index
CEST: Chemical exchange saturation transfer
CRP: C-reactive protein
DMARD: Disease-modifying antirheumatic drug
FoV: Field of view
GAG: Glycosaminoglycan
IVD: Intervertebral disk
MRI: Magnetic resonance imaging
mSASSS: Modified Stoke Ankylosing Spondylitis Spinal Score
MTR_asym_: Magnetization transfer ratio asymmetry
OA: Osteoarthritis
r-axSpA: Radiographic axial spondyloarthritis
SIJ: Sacroiliac joint
SPARCC: Spondyloarthropathy Research Consortium of Canada
STIR: Short tau inversion recovery
TE: Echo time
TR: Repetition time
VC: Vertebral corner
